# Ensemble of vision transformer architectures for efficient Alzheimer’s Disease classification

**DOI:** 10.1186/s40708-024-00238-7

**Published:** 2024-10-03

**Authors:** Noushath Shaffi, Vimbi Viswan, Mufti Mahmud

**Affiliations:** 1 Department of Computer Science, College of Science, Sultan Qaboos University, P.O. Box: 36, Al-Khod, 123 Muscat, Sultanate of Oman; 2 College of Computing and Information Sciences, University of Technology and Applied Sciences, OM 311 Sohar, Sultanate of Oman; 3 Department of Computer Science, Nottingham Trent University, Nottingham, NG11 8NS UK; 4 Medical Technologies Innovation Facility, Nottingham Trent University, Nottingham, NG11 8NS UK; 5 Computing and Informatics Research Centre, Nottingham Trent University, Nottingham, NG11 8NS UK

**Keywords:** Vision transformer, Convolutional neural networks, Machine learning models, Alzheimer’s Disease, Swin transformer, Data efficient image transformers, Bidirectional encoder representation from image transformers

## Abstract

Transformers have dominated the landscape of Natural Language Processing (NLP) and revolutionalized generative AI applications. Vision Transformers (VT) have recently become a new state-of-the-art for computer vision applications. Motivated by the success of VTs in capturing short and long-range dependencies and their ability to handle class imbalance, this paper proposes an ensemble framework of VTs for the efficient classification of Alzheimer’s Disease (AD). The framework consists of four vanilla VTs, and ensembles formed using hard and soft-voting approaches. The proposed model was tested using two popular AD datasets: OASIS and ADNI. The ADNI dataset was employed to assess the models’ efficacy under imbalanced and data-scarce conditions. The ensemble of VT saw an improvement of around 2% compared to individual models. Furthermore, the results are compared with state-of-the-art and custom-built Convolutional Neural Network (CNN) architectures and Machine Learning (ML) models under varying data conditions. The experimental results demonstrated an overall performance gain of 4.14% and 4.72% accuracy over the ML and CNN algorithms, respectively. The study has also identified specific limitations and proposes avenues for future research. The codes used in the study are made publicly available.

## Introduction

Alzheimer’s Disease (AD) is one of the most prevalent and challenging neurodegenerative diseases profoundly impacting individuals, families, and societies worldwide. AD is characterized by the build-up of neuritic plaques and neurofibrillary tangles from amyloid-beta peptide-A in the medial temporal lobe of the brain and neocortical areas [1]. The disease slowly progresses, primarily affecting cognition, memory, and overall brain function. The impact of AD goes beyond cognitive decline and can affect various aspects like memory loss, disorientation, difficulties in problem-solving, and quality of life [2]. The AD continuum has multiple stages. The Mild Cognitive Impairment (MCI) stage is an intermediate phase between cognitive decline due to normal aging and a more noticeable stage of dementia. The Early MCI (EMCI) and Late MCI (LMCI) terms are used to categorize different stages of cognitive impairment between normal cognitive aging and more severe cognitive decline associated with AD. EMCI refers to individuals in the early MCI and may experience noticeable cognitive changes that do not yet significantly impact their daily functioning. EMCI individuals are at an increased risk of developing more severe cognitive impairment or AD. LMCI represents a stage of MCI where cognitive deficits have become more pronounced than EMCI. These individuals may experience more noticeable cognitive difficulties affecting their daily activities and are at a more elevated risk of progression to AD.

As AD progresses, it can impose emotional, financial, and logistical burdens on caregivers and families. Additionally, AD challenges healthcare systems with rising costs and strains on resources due to its increasing prevalence in aging populations – affecting approximately 50 million people across the globe. This number is expected to double every 5 years, with an estimated 152 million cases by 2050 [3]. The recent World Alzheimer’s report [4] emphasizes an imperative need for early detection and intervention by implementing dementia-friendly policies and practices. The use of Artificial Intelligence (AI) research has proven effective in the early detection of AD, thus facilitating early intervention [5].

**Fig. 1:**
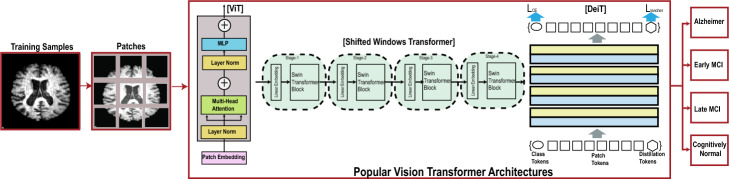
Overview of AD classification using variants of vision transformer architectures

To detect early signs of AD, the AI models can analyze clinical records, neuroimaging scans, and biomarkers [6] to offer significant advancements. Single-classifier based AI models, which are simple and resource-efficient, were the traditional methods used for AD classification. They primarily include different types of machine and deep learning models and feature extraction techniques. However, they face challenges with high-dimensional medical data, leading to sub-optimal accuracy, generalization performance, computational inefficiencies due to large datasets, and sensitivity to data imbalance. They are also prone to overfitting since they may not effectively capture the subtle pattes critical for accurate diagnosis. On the contrary, ensemble methods provide robustness by combining the predictions of multiple models, resulting in more reliable and versatile predictions [5]. Ensembles also reduce the risk of overfitting and make the system adaptable to variations in the data, besides mitigating biases and variances present in individual models, resulting in more reliable and versatile predictions. While traditional classifiers have shown promise in AD detection, recent advancements in VTs offer novel methodologies that could further enhance diagnostic accuracy and provide new insights into AD disease diagnosis [7]. Their ability to capture long-range dependencies and process the sequence tokens in parallel mode contributes to their robust results across benchmark datasets.

The following are prominent reasons to undertake this endeavor:

1. As the field of AI rapidly evolves, it becomes imperative to examine Vanilla VTs’ performance for the task of AD classification both individually and as ensembles. We do not see an exclusive study that does this kind of investigation under both data-scarce and data-rich conditions.
2. Ensemble classifiers combine predictions from multiple classifiers and can achieve higher accuracy and generalization capability. And, to the best of our knowledge, there is no exclusive ensemble ViT study in current literature that utilizes both ADNI and OASIS datasets.
3. The results of VTs and their ensembles were compared with state-of-the-art ML and CNN-centric DL models. Such a comparative study would help both new entrants and real-time domain experts choose the models based on data availability and resources.

In this work, we selected four vanilla VTs to develop the ensemble model using max-voting and probability-based fusion for robust AD classification. The AD classification overview using VT architectures variants is shown in Fig. 1. Specifically, we selected Microsoft’s Shifted window transformer (Swin) [8], Facebook’s Data-efficient Image Transformer (DeIT) [9], BERT pre-trained Image Transformers (BeIT) [10] and Google’s Vision transformers (ViT) [11]. These vanilla VTs were selected based on their diversity in architectural design, proven performance on benchmark datasets, and complementary strengths. Collectively, these models represent a comprehensive and robust ensemble for efficient AD classification tasks.

The rest of the article is organized as follows: The related literature on the use of VT and ensemble is discussed in Sect. 2. The ensemble techniques used with VT are presented in Sect. 3. Experimental results and analysis are discussed in Sect. 4, Challenges and future avenues are covered in Sect. 5, and Conclusions are drawn in Sect. 6.

## Related work

Application of AI inspired by the success of Transformers in NLP, VTs are now being widely used in medical imaging, specifically for analyzing neuroimaging data [12]. They show promise in early detection and diagnosis, especially with various neuroimaging modalities like MRI. The attention mechanism of VTs plays a key role in capturing complex patterns and dependencies in brain images. This section provides a review of literature that uses VTs in the classification of AD.

Maram et al. [13] applies the ViT approach to detect AD using MRI images, showcasing good accuracy and precision on the OASIS dataset. The model focuses on critical regions demonstrating high accuracy, precision, recall, F1-score, and minimal false negatives. The study shows that the ViT model performs well. Using the OASIS dataset for AD, Sherwani et al. [14] compare the effectiveness of CNN and VT using two variants of VT architectures: Deep ViT and Class attention in Image Transformer (CaiT) [15]. While Deep ViT achieved an accuracy of 90.2%, CNN achieved an accuracy of 82.0% only.

Odusami et al. [16] combine MRI and PET datasets from the ADNI repository using discrete wavelet transform (DWT) and apply transfer learning using the pre-trained neural network VGG16. An inverse discrete wavelet transform is used to reconstruct the fused image, and then a ViT that has already been trained is used to classify it. The study showed accuracy of over 98% for fused data when compared to 94% for CNN. Another study by Lyu et al. [17] introduces a cross-domain transfer learning approach to address data insufficiency using ViT for AD vs CN classification pre-trained on the ImageNet-21K dataset and fine-tuned on the brain imaging dataset. The incorporation of a slice-wise convolution embedding enhances the methodology. The results show that ViT outperforms CNN-based architecture and effectively transfers knowledge. Saman et al. [18] introduce a robust and optimized VT architecture for classifying healthy controls, MCI and AD using resting-state functional MRI (rs-fMRI) and structural MRI (sMRI) data from ADNI. The study also uses Deep ViT with a re-attention mechanism and class-attention in image transformers (CaIT). The model, with 30% fewer parameters than a vanilla transformer, achieved F1-scores of 97% and 99.55% for rs-fMRI and sMRI modalities, respectively.

Besides using datasets from ADNI, the authors in [19] have compared the performances of AD detection for datasets from Australian Imaging Biomarkers and Lifestyle Study (AIBL). In this work, authors propose a variant of ViT, namely, Resize Swin Transformer (RST), to address the challenges of brain imaging due to limited dataset sizes and labor-intensive preprocessing procedures. This model extracts information from processed brain images, achieving multi-scale and cross-channel features. The RST is pre-trained on a natural image dataset and demonstrates superior classification performance with over 99% and 94% accuracy on ADNI and AIBL datasets. The performances are compared with CNN-based models, namely DenseCNN, CNN and ResNet. The ViT variant exhibits better classification performance in AD prediction. Another study by Shin et al. [20] proposes a ViT approach for classifying AD using 18F-Florbetaben brain imaging, addressing the need for accurate diagnostic tools for early detection and treatment of AD. The method uses the ViT architecture to process PET scans that reveal amyloid plaque accumulation, a key indicator of AD. The implementation involves preprocessing the images to enhance quality before training the model. Performance evaluation demonstrates that the ViT approach significantly outperforms traditional methods in classifying AD versus non-AD subjects. The advantages of this approach include its ability to capture complex spatial relationships in imaging data and robust performance with multimodal inputs. Overall, the findings suggest that ViT can be a powerful tool in the clinical diagnosis of AD.

Several studies use an ensemble framework of VT and CNN. For instance, Rahma et al. [21] introduce two methods for AD diagnosis. The first method combines a Swin transformer with an enhanced EfficientNet, multi-head attention, and a Depthwise Over-Parameterized Convolutional Layer (DO-Conv). The second method modifies the CoAtNet (a family of hybrid models - depthwise Convolution and Self-attention) network with ECA-Net (Efficient Channel Attention network) and fused inverted residual blocks. Evaluation of the OASIS and ADNI datasets, along with Grad-CAM analysis, yields promising results. The former method achieves 93.2% accuracy on OASIS, while the latter method achieves 97.33%. Similarly, the study of Nanni et al. [22] fuses CNN with transformer ensembles, including Deit, ViT, and Swin topologies. The experimental results across multiple datasets show significant performance improvements when combining diverse models for image classification tasks. In another article, Chen et al. [23] propose an ensemble of CNN and VT in predicting AD stages, effectively differentiating between healthy controls, very mild, mild, and moderate cases. The ensemble model achieves good classification accuracy. Xing et al. [24] introduce a ViT model trained on multimodal PET images from ADNI followed by classification for AD diagnosis achieving an accuracy of 91% and an AUC of 0.95.

**Table 1:** Key findings of the related literature

Ref	Objective	Methodology	Dataset	Results
[13]	ViT approach to detect AD	Attention-based mechanism	Kaggle	99% Precision, Recall, and F1-Score
[14]	Comparing the effectiveness of CNN and VT in AD detection	Deep ViT and Class attention in image Transformer (CaiT)	OASIS	ViT accuracy 90.2% CNN accuracy 82%
[16]	To analyze fused datasets of MRI and PET, train using VGG16, and classify using pretrained ViT	Discrete Wavelet Transform (DWT) for fusing, training with VGG16, and classifying using pretrained ViT	ADNI	ViT accuracy 98.0% CNN accuracy 94.0%
[17]	Cross domain transfer learning approach to address data insufficiency in brain image data	ViT pretrained on ImageNet-21K and CNN based architecture	ADNI	ViT outperforms CNN based architecture
[18]	Using ViT for AD classification using rs-fMRI and sMRI	Deep ViT with re-attention mechanism and Class attention in image Transformer (CaiT)	ADNI	F1-Score of 97% for rs-fMRI and 99.55% for sMRI
[19]	Using ViT to address challenges of brain images due to limited dataset sizes and labor-intensive preprocessing procedures and compare with DenseCNN, CNN and ResNet	Variant of ViT called Resize Swin Transformer (RST)	ADNI and AIBL	Over 99% an 94% accuracy on ADNI and AIBL datasets
[20]	Classifying AD using 18F-Florbetaben brain imaging and leveraging the ViT architecture	ViT architecture to process PET scans that reveal amyloid plaque accumulation	Multicite Dong-A University cohort	ViT showed better performance (51.74%) than VGG (36.73%) when using augmented data
[21]	AD classification using robust VT transformer methods - Swin and a Depthwise Over-Parameterized Convolutional Layer (DO-Conv)	Swin transformer with enhanced EfficientNet multi-head attention and Depthwise Over-Parameterized Convolutional Layer that modifies the CoAtNet network with ECA-Net and fused inverted residual block	ADNI and OASIS	Swin achieves 93.2% accuracy and DO-Conv achieves 97.33% on the OASIS dataset
[22]	Presents an automated image classification system for AD that fuses CNN with ViT transformer ensembles	CNN and transformer architectures - Deit, ViT, and Swin topologies	Multisite and Kaggle	Results across multiple datasets show significant performance improvements when combining diverse models for image classification tasks
[23]	Presents an ensemble of CNN and VT in predicting AD, effectively differentiating between healthy controls, very mild, mild, and moderate cases	CNN (ResNet50, DenseNet121, EfficientNetB0) and Transformer Architectures (ViT and TransFG)	OASIS	Ensemble model exhibits an accuracy, with 98.91%, a Micro Area Under the Curve (micro-AUC) of 0.9996, and a macro-AUC of 0.9995
[24]	Classification for AD diagnosis using Vision transformer models trained on multimoal PET images	ViT model trained on multi-modalities of PET and PET-FDG	ADNI	Outperforms several strong baseline models and achieves 0.91% accuracy and 0.95 AUC

This review of relevant literature, summarized in Table 1, suggests a widespread adoption of VTs in AD classification. While several studies have investigated the integration of VTs with CNNs, research exclusively dedicated to ensembles of vanilla VTs is unavailable for AD classification. Table 1 also shows models with several gaps that must be addressed. Common gaps like high computational demands and the need for large datasets are found in VTs. To mitigate these gaps, the proposed work fine-tune the VT models and incorporate diverse datasets from both ADNI and OASIS that will enhance generalizability and clinical applicability. Additionally, exploring advanced ensemble techniques can improve the model performance and reliability. This study aims to address these significant gaps by proposing an ensemble of Vanilla VTs and testing their performance using OASIS and ADNI datasets, thereby also providing a comparative evaluation with CNN-centric architectures. We also observed that no exclusive study exists on the comparative evaluation of VTs against the popular CNN architectures that utilize both ADNI and OASIS datasets.

## Ensemble of vision transformers

**Fig. 2:**
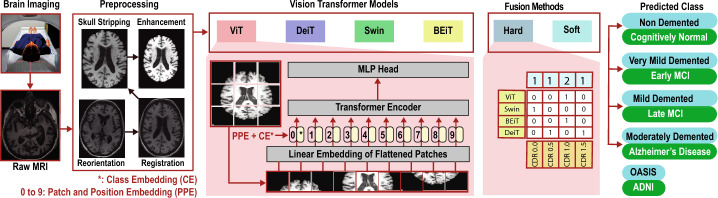
The proposed pipeline and its main components: (i) preprocessing and (ii) vision transformer models, namely, Google’s Vision Transformers (ViT) [11], Data-Efficient Image Transformer (DeiT) [9], Shifted windows Transformer (Swin) [8], and Bidirectional Encoder Representation from Image Transformers (BEiT) [10]. The detailed working mechanism of ViT is adapted from [11]. There are four vision transformer models to choose from that have been tested with the OASIS and the ADNI datasets. (iii) Decision Level Fusion, namely Hard-voting and Soft-voting. The hard voting mechanism is shown. (iv) AD Classification Labels, OASIS (Blue) and ADNI (Green)

This section aims to provide a complete pathway of how an MRI image is inferenced through an ensemble of VT framework for a robust AD classification. Figure 2 shows the proposed pipeline depicting the three main components, namely, (i) preprocessing of brain images (covered in section 3.1), (ii) VT model training (covered in section 3.2), and (iii) ablation study of vanilla transformers elaborated in section 3.3.

### MRI preprocessing

**Fig. 3:**
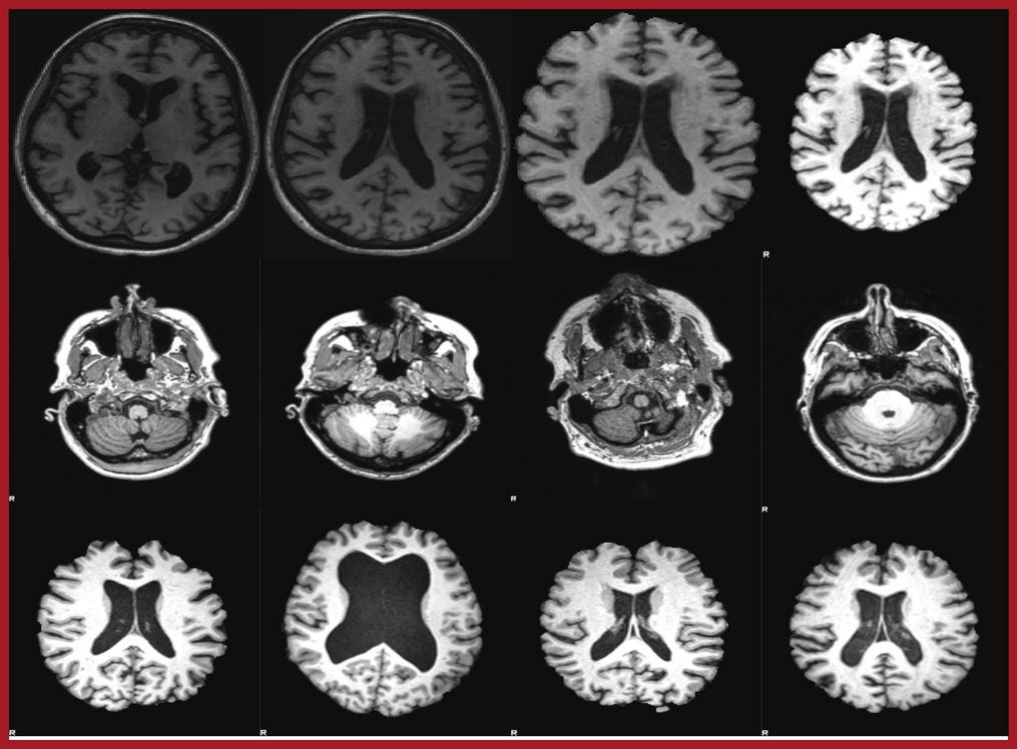
First Row – Intermediate Stages of Preprocessing: (i) reorientation, (ii) registration, (iii) skull-stripping, (iv) histogram equalisation. Second Row – Sample raw MRI scans from the ADNI cohorts. Third Row – Corresponding final preprocessed output

The MRI images downloaded from ADNI contained raw data, and hence, they had to be preprocessed. The downloaded MRI files underwent four preprocessing stages: i) reorientation, ii) registration, iii) skull-stripping, and iv) histogram equalisation. The reorientation step aligns the brain images to MNI standard space where anterior portion of the brain is upward facing while the superior side is forward facing. This step ensures correction of any orientation variation due to differences in scanner settings or patient positioning during the scan. The registration process ensures alignment of brain images from different subjects or time points into a common coordinate space. This alignment is crucial for comparing and analyzing images across individuals or longitudinal studies. The skull-stripping step is done to eliminate artifacts and non-brain structures such as skull, scalp, and extracranial tissues which are not useful in the AI based classification of brain images. In the final step histogram equalization enhances MRI features in the brain images. The sample images from this preprocessing process are detailed in Fig. 3

### Overview of vision transformers used in the study

In light of the emergence of ViT [11], there have been significant advancements in VT architecture in recent times. Several prominent VT variants have come to fore, namely BEiT [10], Swin Transformers [8], and DeiT [9]. These architectures have demonstrated remarkable performance across various benchmarks, including ImageNet, compared to contemporary CNNs. In this section, we provide a brief overview of the VT variants.

#### Vision trasformer (ViT)

Image patches are fed as 1D vectors to the standard Transformer Encoder (TE) of the ViT [11]. The input image of dimension $x \in {\mathbb {R}}^{H \times W \times C}$ is divided into a series of 2D patches $x_p \in {\mathbb {R}}^{(N \times (P^{2} \times C))}$ – where (*H, W*) represents the original image dimension, *C* denotes the number of channels, *(P, P)* denotes the patch dimension. The total number of patches obtained is determined using $N=HW/P^{2}$. The image patches are transformed to patch embeddings of *D* dimension. The positional encodings and learnable class token ($z_0^0 \in x_{class}$) are appended to these embeddings.

1 $$\begin{aligned} z'_l= & MSA(LN(z_{l-1}) + z_{l-1}, \quad where \quad l = 1...L \end{aligned}$$

2 $$\begin{aligned} z_l= & MLP(LN(z'_{l}) + z'_{l}, \quad where \quad l = 1...L \end{aligned}$$

These embedding vectors sequence serves as input to the TE. The TE consists of alternating layers of Multiheaded Self-Attention (MSA) and Multi-Layer Perceptron (MLP) blocks. The layer normalization (LN) process is performed before and after each block’s residual connections. Equations 1 and 2 represent MSA and MLP along with LN. The MLP head finally provides the class label of the inference image.

#### Data-efficient image transformer (DeiT)

Touvron et al. [9] introduced DeiT to mitigate ViT’s reliance on extensive datasets. DeiT facilitates ViT training on smaller datasets such as ImageNet-1k and employs a teacher-student framework to train a more compact transformer model. A new distillation token was introduced to interact with the class and patch tokens in addition to the token utilized by ViT. Distillation can be categorized into two types: soft and hard. The teacher model supplies probability distributions in soft distillation as “soft” labels. In hard distillation, “hard” labels are the class predictions or decisions the teacher model provides. The difference between the outputs of the teacher and student models is minimized in soft distillation using Equation 3.

3 $$\begin{aligned} \begin{aligned} {\mathcal {L}}_{global}&= (1-\lambda ){\mathcal {L}}_{CE}(\psi (Z_s),y)\\&\quad +\lambda \tau ^{2}KL(\psi (Z_s/\tau ),\psi (Z_t/\tau )) \end{aligned} \end{aligned}$$

In hard distillation, the teacher’s decision is taken as the true label. Equation 4 yields the hard distillation using the teacher’s true label, $y_t = argmax_cZ_t(c)$.

4 $$\begin{aligned} {\mathcal {L}}_{global}^{hardDistill} = \frac{1}{2}{\mathcal {L}}_{CE}(\psi (Z_s),y)+\frac{1}{2}{\mathcal {L}}_{CE}(\psi (Z_s),y_t) \end{aligned}$$

Soft and hard distillation are training methods used to transfer knowledge from a larger teacher model to a smaller student model. The selection between soft and hard distillation hinges on the model’s specific objectives and the desired characteristics in the final predictions. These strategies aim to enhance the predictions of the student model by utilizing the knowledge distilled from a larger teacher model. Once the distillation process is complete, the student model can autonomously predict new, unseen data. Essentially, the distillation process represents a form of transfer learning where the student model acquires knowledge from the teacher model. The final classification utilizes only the trained student model, which independently makes predictions following the knowledge transfer process.

#### Shifted windows transformer (Swin)

The Swin Transformer, introduced by Liu et al. [8], represents an evolution of the Transformer block, replacing the traditional multi-head self-attention (MSA) module with a shifted window-based module. The Swin Transformer block is structured with a shifted window-based MSA module, supplemented by an MLP module. It includes LN layers positioned before each MSA module and MLP, along with residual connections after each module. In Swin, a “window” denotes a partition or block of the input image that undergoes separate processing within the self-attention mechanism. The Swin Transformer employs a hierarchical architecture that divides the input image into non-overlapping patches or windows at multiple scales or levels. Within each window, self-attention is applied independently, allowing the model to capture both local and global dependencies effectively across different spatial resolutions.

For large images, window-based self-attention provides scalability and efficiency, making it a preferred choice. The Swin Transformer utilizes an approach that alternates between non-overlapping window partitioning and shifted window partitioning configurations within its blocks. This strategy optimizes the model’s ability to capture spatial relationships across varying scales effectively. The initial layer employs a standard partitioning strategy, evenly dividing the window into *M* x *M* blocks. The subsequent layer utilizes a shifted configuration, displacing the windows by ($\lfloor \frac{M}{2}\rfloor$, $\lfloor \frac{M}{2}\rfloor$) pixels from the regularly partitioned windows in the previous layer. In this approach of partitioning, consecutive Swin Transformer blocks are computed as shown in equation 5.

5 $$\begin{aligned} \begin{aligned} {\hat{z}}^{l}&= W-MSA(LN(z^{l-1}))+z^{l-1},\\ {z}^{l}&= MLP(LN({\hat{z}}^{l}))+{\hat{z}}^{l},\\ {\hat{z}}^{l+1}&= SW-MSA(LN(z^{l}))+z^{l},\\ {z}^{l+1}&= MLP(LN({\hat{z}}^{l+1}))+{\hat{z}}^{l+1}, \end{aligned} \end{aligned}$$

where ${\hat{z}}^{l}$ and $z^l$ represents the output features of the $SW-MSA$ and the *MLP* module for block *l* respectively. $W-MSA$ and $SW-MSA$ indicate window-based multi-head self-attention using shifted window partition configurations.

#### Bidirectional encoder representation from image transformers (BEiT)

Inspired by the success of Bidirectional Encoder Representation from Transformers (BERT) in NLP, Bao et al. [10] proposed the BEiT VT model. The BEiT model is pre-trained using BERT’s masked image modeling (MIM) framework, which utilizes two input views for each image: *image patches* and *visual tokens*.

The BEiT transforms the 2D image $x \in {\mathbb {R}}^{H\times W \times C}$ into $N = HW/P^2$ patches $x^p \in {\mathbb {R}}^{N\times (P^2C)}$. Here, *C* represents the number of channels, (*H*, *W*) denotes the input image resolution, and (*P*, *P*) is the resolution of each patch. The raw image patches $\{x_i^p\}^N_{i=1}$ are vectorised to be used as input features. The tokenization step, through the discrete variational autoencoder latent codes, transforms the image into visual tokens [25].

Approximately 40% of the image patches undergo random masking in the MIM framework. The positions that are masked are represented as $M \in \{1,\cdots , N\}^{0.4N}$. The patches that are masked are subsequently substituted with a learnable embedding $e_{[M]} \in {\mathbb {R}}^D$. The Transformer block will then be provided with corrupted image patches $x^M = \{x^p_i: i \notin M\}^N_{i=1} \bigcup \{e_{[M]}: i \in M\}^N_{i=1}$ as inputs. The final hidden vectors $\{h^L_i\}^N_{i=1}$ are considered to be the encodings of the input patches. The softmax classifier is used at each masked position $\{h^L_i: i \in M\}^N_{i=1}$ to predict the corresponding visual tokens $p_{MIM}(z'|x^M) = softmax_{z'}(W_ch^L_i+b_c)$, where $x^M$ is the corrupted image, $W_c \in {\mathbb {R}}^{|V|\times D}$, and $b_c \in {\mathbb {R}}^{|V|}$.

### Ensemble techniques

The essence of ensemble classification is to improve prediction performance by fusing the predictions of multiple individual classifiers. Our study used two common fusion approaches: Max voting and probability-based voting, also termed hard voting and soft voting. This section describes the two fusion techniques used in our study.

#### Max-voting-based fusion (Hard voting)

Max or Hard voting is a prediction technique generally used for classification problems. For the given set of *n* models, with prediction vectors $\overrightarrow{p}_i (i=1, \cdots , n)$. The prediction vectors are also termed as *vote*. The final prediction is determined by finding the most commonly predicted label by taking the mode of the predicted labels among the individual classifiers or by considering the highest *vote* obtained from majority of the models [5, 26].

6 $$\begin{aligned} P_{MV} = mode (\overrightarrow{p}_{i}) \end{aligned}$$

#### Probability-based ensemble (Soft voting)

Probability based ensemble or Soft voting is an effective algorithm that combines the predictions of multiple classifiers by utilizing the probability of predictions. In this approach probabilities are assigned to each class for each classifier. The class with the highest total probability is considered as the prediction of the ensemble [5, 26]..

Let $\beta _{k}^{i} (i=1,\cdots ,n, k=1,\cdots ,C)$ represent the probability output of n models belonging to *C* classes. This ensemble technique predicts the final label by summing the predicted probabilities by individual models.

7 $$\begin{aligned} Pl^j = \mathop {\textrm{argmax}}\nolimits _{{\textrm{l}} \in {\textrm{C}}} \left( \sum _{i=1}^{n} \beta _1^{i},\sum _{i=1}^{n} \beta _2^{i}, \cdots , \sum _{i=1}^{n} \beta _C^{i}\right) \end{aligned}$$

## Results

### Experimental setup

The details pertaining to the experimental setup such as the number of samples, train-test subsets, preprocessing pipeline, hyper-parameters, etc. are all described in this section.

#### Dataset

The experimentation was conducted using Open Access Series of Imaging Studies (OASIS, www.oasis-brains.org) and Alzheimer’s Disease Neuroimaging Initiative (ADNI, https://adni.loni.usc.edu/) datasets.

*The OASIS dataset:* This dataset consists of 6400 images classified into four different classes based on Clinical Dementia Rating (CDR) score. The classes *non-demented*(3200), *very mild demented*(2240), *mild demented*(896) and *moderately demented*(64) maps to CDR score of 0, 0.5, 1.0 and 2.0 respectively. The values in the parenthesis denote the total samples in the respective classes. The dataset was divided into 70:30 train-test subsets with 5120 and 1280 samples, respectively. The preprocessed image files suitable for processing was downloaded from the Hugging Face platform [27].

*The ADNI dataset:* T1-weighted MRI images with Magnetisation Prepared - RApid Gradient Echo (MPRAGE) from subjects belonging to either genders aged between 50 and 65 were downloaded. The MPRAGE technique is used to highlight the anatomical structure of gray and white matters in the brain region [28]. We downloaded 1056 raw MRI images from the Axial plan belonging to four categories: AD (223), EMCI (475), LMCI (262), and CN (96), resulting in 1056 samples. The total number of samples in each class is denoted in parentheses. Train and test samples were split into 90:10 proportions, with 950 training and 106 test samples to ensure that the model has enough data to learn from. Images in this dataset are taken from ADNI-1, ADNI-2, and ADNI-GO cohorts and downloaded in NIfTI format. The dimension of ADNI MRI image used in our experimentation is $218 \times 192$.

#### Metrics

The evaluation of models involved the utilisation of accuracy($A_{c}$), sensitivity($S_{e}$), and specificity($S_{p}$) as performance metrics [5]. Accuracy measures the ratio of correctly classified samples to the total number of samples. Specificity($S_{p}$), or the true negative rate (TNR), quantifies the test’s ability to correctly identify individuals without the specific ailment being tested. The false positive rate (FPR) can be derived as $1-S_{p}$. Sensitivity($S_{e}$), also referred to as recall or the true positive rate (TPR), evaluates the test’s ability to accurately identify individuals with the targeted ailment. The false negative rate (FNR) can be calculated as $1-S_{e}$. All reported values were averaged using the one-vs-all strategy.

#### Model implementation

The pretrained weights of the VT models were downloaded from the Hugging Face platform. The pretrained models utilized were all the Vanilla version of Google’s ViT, Microsoft’s Swin and BeiT, and Facebook’s DeiT models. The ViT, Swin and BeiT were trained using ImageNet’s 21K dataset (14 million samples spanning 21,841 classes). The DeiT model, known as a data efficient model, was pretrained utilizing ImageNet’s 1K dataset. The ML models were implemented using the well known Python library Scikit-learn. The pretrained CNN models were obtained from the Tensorflow package. The fine-tuning and subsequent evaluation was carried out on a Windows system equipped with NVidia RTX 3060 GPU and 3.2 GHz CPU. The code used in this study can be downloaded at: https://github.com/snoushath/VIT-CNN-AD.git.

### Model training

**Fig. 4:**
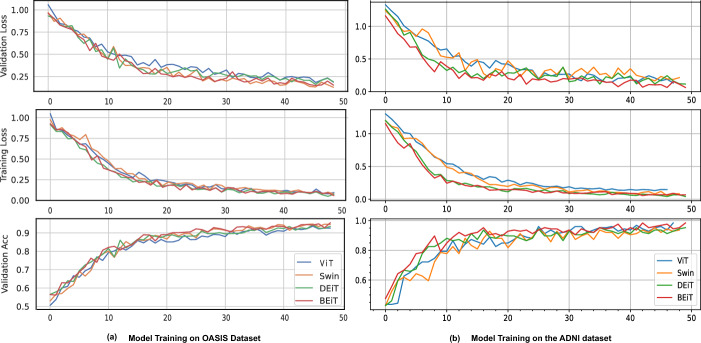
Model Training of Vanilla VT algorithms: **a** On OASIS Dataset, **b** On ADNI Dataset

In this experiment, we analyze the training dynamics of four different VT architectures using both OASIS and ADNI datasets. As mentioned previously, the OASIS represents a balanced dataset with sufficient samples and the ADNI dataset represents a data-scarce condition. We represent and compare the distribution of validation loss, training loss, and validation accuracy across 50 epochs using line plots as shown in Fig. 4.

The line plots of all four algorithms (on both OASIS and ADNI datasets) depict a consistent downward trend in both validation and training loss, suggesting the efficacy of VT models in capturing AD patterns during fine-tuning. Moreover, the observed increase in validation accuracy across all algorithms signifies successful generalization to the validation data. Notably, despite being trained on a smaller ImageNet-1K dataset, the fine-tuned DeiT model demonstrates comparable trends with the other three models which were pretrained on a significantly larger ImageNet-21K dataset. This demonstrates the DeiT’s data-efficient capability to train from a smaller pretraining dataset.The observed trends in performance remain consistent across models, irrespective of whether the fine-tuning was conducted using a more balanced dataset (OASIS) or an imbalanced and smaller dataset (ADNI). This consistency shows the robustness and generalization capabilities of the VT models across varying data conditions. Despite the inherent differences in dataset characteristics, including size and class distribution, the VT models exhibit comparable effectiveness in capturing AD patterns and achieving successful generalization during the fine-tuning process.

**Table 2:** Performance of VTs on various performance metrics on OASIS and ADNI datasets

Models	OASIS dataset					ADNI dataset				
	Acc	Spe	Sen	FNR	FPR	Acc	Spe	Sen	FNR	FPR
ViT	0.9718	0.9882	0.91915	0.0808	0.0118	0.9904	0.9963	0.9919	0.0080	0.0036
Swin	0.9804	0.9913	0.9415	0.0584	0.0086	0.9952	0.9963	0.9887	0.01124	0.00368
DeiT	0.9843	0.9931	0.9696	0.0303	0.0068	0.9904	0.9963	0.9887	0.0112	0.0036
BEiT	0.9773	0.9905	0.9533	0.0466	0.0094	0.9904	0.9963	0.9887	0.0112	0.0036

### Model testing

In this experiment, we used both OASIS and ADNI datasets to check the efficacy of trained vanilla VTs under both data-sufficient (OASIS) and data-constrained (ADNI) situations.

The evaluation results, presented in Table 2, demonstrate an outstanding performance by all models, with comparable test accuracies. The table reveals that across all models, including ViT, Swin Transformer, DeiT, and BEiT, remarkable accuracy is achieved on the OASIS dataset, ranging from 97.18% to 98.43%. Notably, both specificity and sensitivity values exhibit consistency, signifying robust performance in correctly identifying true negatives and true positives. Additionally, the low FNR and FPR indicate the models’ ability to minimize classification errors effectively. Similarly, the models exhibit commendable performance on the smaller ADNI dataset, with accuracy ranging from 99.04% to 99.52%. Specificity and sensitivity values remain consistently high, indicative of the models’ efficacy in accurately classifying both inter-class and intra-class cases within the dataset.

It is important to note that despite the ADNI dataset’s smaller size relative to OASIS, the VT models maintain comparable or even slightly higher accuracy on ADNI, highlighting their ability to fine-tune with limited samples and generalize effectively to smaller datasets. This feature is critical for medical scenario applications where data availability may be limited. This ability of VT models to effectively learn from and adapt to smaller datasets underscores their robustness and generalization capabilities. The VT models’ consistent performance across datasets indicates their adaptability to fine-tune regardless of whether the underlying dataset is balanced, imbalanced, limited or adequate.

### Fusion of VTs

**Fig. 5:**
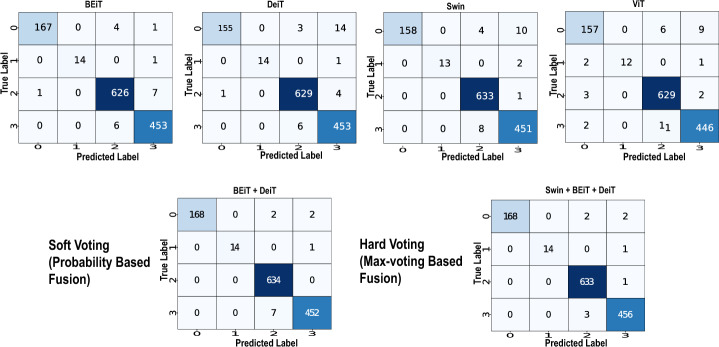
Confusion matrices of vanilla VTs and best ensembles (soft and Hard)

This section reports the results of the ensemble classification of Vanilla VTs. For this purpose, we have considered hard and soft ensemble techniques as described in Section 3. Ensemble classifiers offer several advantages in machine learning [5]: (i) the diversity of algorithms ensures that particular weakness of a classifier is mitigated through a robust classifier in the ensemble, (ii) by combining multiple models, ensembles can achieve higher accuracy and robustness compared to individual models, and additionally (iii) ensemble methods provide stability across different datasets and conditions, generalize well to new data. Here, we provide classification performance of four vanilla VTs as an ensemble of 2, 3, and 4 classifiers and provide substantiated analysis (See Table 3). We used the OASIS dataset only in this experiment as individual model performance on the ADNI dataset was almost 100% (Fig. 5).

Some observations from this experiment are:

1. Firstly, it is to be noted that the ensemble of VT achieved an accuracy improvement of 0.63% to 0.86% compared to individual VT performance. While this increment seems less significant from an academic point of view, its clinical significance can be profound. In a clinical context, even a slighter improvement in accuracy means early diagnosis of AD and the potential for personalized interventions, thereby significantly improving patient outcomes.
2. The enhanced performance of the ensemble of BEiT and DeiT could be due to fundamentally different architectural designs and training objectives offered by these algorithms.
3. BEiT’s architecture extends the transformer architecture by introducing bidirectional encoders, allowing the model to capture local and global contextual information within the image. This bidirectional processing enables BEiT to better understand the relationships between image patches. DeiT focuses on knowledge distillation to distill knowledge from large-scale teacher models into smaller student models. By effectively transferring knowledge from pre-trained models, DeiT achieves competitive performance with significantly reduced computational resources and data requirements.
4. In terms of learning objectives offered, DeiT learns from a teacher model through knowledge distillation, while BEiT learns representations through self-supervised learning. By fusing these models, the ensemble benefits from the diverse learning objectives pursued by DeiT and BEiT.
5. We also noticed that the hard-voting scheme outperformed soft-voting ensembles in cases with more than two classifiers in the ensemble. For example, consider models A, B, and C with probability values [0.2, 0.59, 0.1, 0.01], [0.6, 0.20, 0.1, 0.1], and [0.3, 0.31, 0.2, 0.19], respectively, with ground truth label as 1. Model A predicts class 1, model B predicts class 0, and model C predicts class 1. From this example, model C shows that the probability values among class-0 and class-1 have very narrow differences affecting the dominant decision shown by models A and B. Due to this ambiguity, the sum of probability values exhibits similar confidence between class-0 and class-1 based on soft voting, leading to uncertainty in the decisions. However, the hard-voting technique correctly predicts the final label as class-1 based on the max-voting, outperforming the soft-voting mechanism.
6. DeiT performs better than other VTs despite being trained on smaller datasets primarily due to the process of knowledge distillation. In this process, a larger pre-trained model (typically a ViT) acts as a “teacher” model and transfers its knowledge to a smaller student model (the DeiT). During training, the student model learns to mimic the predictions of the teacher model. This process enables the DeiT to benefit from the rich representations learned by the larger model, even though it is trained on smaller datasets.
7. Furthermore, DeiT typically has fewer parameters compared to ViT due to its smaller size. This parameter efficiency allows DeiT to learn more effectively from the limited data available, leading to better utilization of the dataset and improved performance.
8. Increasing the number of classifiers in the ensemble may not improve the overall accuracy significantly. If the individual classifiers in the ensemble make similar errors on the same data instances, then combining them through majority voting/hard-voting might not lead to improvements in accuracy. Ensembles mainly benefit if presented with diverse type of individual classifiers, meaning each classifier should bring unique perspectives or capture different aspects of the data.

**Table 3:** Performance evaluation of various ensembles of vision transformers

No.of Models	Ensemble	Type	Accuracy	Specificity	Sensitivity	FNR	FPR
2	ViT+Swin	Hard	0.9757	0.9894	0.9297	0.0702	0.0105
		Soft	0.9851	0.9933	0.9484	0.0515	0.0066
	ViT+BEiT	Hard	0.9781	0.9906	0.9333	0.0666	0.0093
		Soft	0.9835	0.9926	0.9638	0.0361	0.0073
	ViT+DeiT	Hard	0.9750	0.9894	0.9276	0.0723	0.0105
		Soft	0.9835	0.9931	0.9457	0.0542	0.0068
	Swin+BEiT	Hard	0.9875	0.9940	0.9725	0.0274	0.0059
		Soft	0.9875	0.9943	0.9531	0.0468	0.0056
	Swin+DeiT	Hard	0.9851	0.9930	0.9701	0.0298	0.0069
		Soft	0.9890	0.9951	0.9539	0.0460	0.0048
	BEiT+DeiT	Hard	0.9859	0.9935	0.9698	0.0301	0.0064
		Soft	0.9906	0.9958	0.9721	0.0270	0.0041
3	ViT+Swin+BEiT	Hard	0.9898	0.9954	0.9552	0.0447	0.0044
		Soft	0.9851	0.9933	0.9484	0.0515	0.0066
	ViT+Swin+DeiT	Hard	0.9851	0.9936	0.9486	0.0513	0.0063
		Soft	0.9851	0.9933	0.9484	0.0515	0.0066
	ViT+BEiT+DeiT	Hard	0.9882	0.9947	0.9685	0.0314	0.0052
		Soft	0.9835	0.9926	0.9638	0.0361	0.0073
	Swin+BEiT+DeiT	Hard	0.9929	0.9968	0.9754	0.0245	0.0031
		Soft	0.9828	0.9923	0.9450	0.0549	0.0076
4	ViT+Swin+BEiT+DeiT	Hard	0.9906	0.9956	0.9737	0.0262	0.0043
		Soft	0.9835	0.9926	0.9464	0.0535	0.0073

### Comparison with CNN

Despite VTs’ advancement in the last few years, CNNs continue to remain the cornerstone of computer vision tasks, mainly for data-efficient architecture training. VTs, on the other hand, require a massive dataset for training from scratch. In this section, we provide a comparative analysis of VTs with CNNs using both ADNI and OASIS datasets. We considered popular EfficientNetB0, InceptionResNetV2, InceptionV3, ResNet50, ResNet101, and ResNet152 pre-trained models for this purpose. The fine-tuning was performed by replacing the last classification layer of these architectures with the following sequence of layers as in [5]:$DO(0.5)-Flatten-BN-2048N-BN-DO(0.5)-1024N-BN-DO(0.5)-4N$. Here, *DO*(*i*) denotes a dropout layer of probability *i*, *BN* implies the Batch Normalization layer and *cN* indicates a fully connected layer consisting of *c* neurons. We empirically fixed a learning rate of $1e10-5$ using the Adam activation function. The VT and CNN models were fine-tuned for 50 epochs. Additionally, popular CNN architectures [29–31] from literature are also used in this comparative study. AlzheimerNet [31] uses an InceptionV3 pre-trained architecture with an RMSProp optimizer and a learning rate of 0.00001. Yousry et al. [29] use a custom-built CNN with an Adam optimizer, where feature maps are initialized using a Glorot initializer. A study by Helaly et al. [30] utilizes the VGG19 pre-trained architecture. The results are reflected in Tables 4 and 5.

#### Using the OASIS dataset

This section reports the performance of VTs with various CNN models using the OASIS dataset.

All four vanilla VT models performed well as indicated by their training and validation losses. These models are effective in capturing the underlying AD patterns well in the MRI samples which is evident in their generalization ability as indicated by their test accuracies. However, the popular EfficientNetB0 architecture exhibited high training and validation errors. The large gap in their training and validation error indicates that the model is overfitting the data as its test accuracy is only 0.4380. InceptionResNetV2 and InceptionV3 models also exhibit overfitting behavior as their validation losses are high compared to training losses. For instance, InceptionV3 has a notable difference in test (0.73) and validation (0.93) accuracy. The CNNs do not exhibit any signs of underfitting based on the results in Table 4. All variants of ResNet architectures demonstrated good performance without any signs of overfitting or underfitting which is evident through their high test and validation accuracy. Overall, the VTs demonstrated robust performance as against CNN models.

**Table 4:** Vision transformers vs CNNs on OASIS dataset

Architectures	Test	Val	Train	Val
	Acc	Acc	Loss	Loss
EfficientNetB0	0.4380	0.9259	0.7412	0.6825
InceptionResNetV2	0.5210	0.8144	1.0295	0.9748
InceptionV3	0.7312	0.9329	0.5748	0.6860
ResNet50	0.8492	0.9659	0.3030	0.4474
ResNet101	0.8296	0.9640	0.3074	0.4510
ResNet152	0.8554	0.9711	0.2919	0.4024
Shamrat et.al [31]	0.8695	0.9635	0.2897	0.3698
Helaly et.al [30]	0.8898	0.9563	0.3358	0.3581
Yousry et. al [29]	0.9012	0.9536	0.3025	0.3231
DeiT	**0**.**9843**	**0**.**9550**	**0**.**0570**	**0**.**1483**

#### Using the ADNI dataset

In this experiment, we use a smaller subset of the ADNI dataset to assess the effectiveness of VTs in learning and generalizing from limited samples. The outcomes of this experiment are summarized in Table 5.

The performance analysis conducted on the smaller ADNI dataset highlights the efficiency of Vision Transformers (VTs) when compared to Convolutional Neural Networks (CNNs). Particularly noteworthy are models like BEiT, ViT, and Swin, which showcase remarkable adaptability despite the constraints of limited data. The results reveal consistently high accuracy and relatively low loss values for VTs, indicating their ability to effectively learn from smaller datasets. Notably, BEiT stands out with a remarkable 100% accuracy on the test set, underscoring its exceptional fine-tuning capability even with limited samples. In contrast, certain CNNs, such as EfficientNetB0 and ResNet152, exhibit signs of potential overfitting, as evidenced by larger disparities between test and validation accuracies. This disparity emphasizes the robustness of VTs in mitigating overfitting challenges, making them particularly advantageous in scenarios characterized by limited data availability, such as medical health contexts. Overall, this experiment underscores the superior performance and generalization capabilities of VTs, even in scenarios with restricted training examples.

**Table 5:** Vision transformers vs CNNs on ADNI dataset

Architectures	Test	Val	Train	Val
	Acc	Acc	Loss	Loss
EfficientNetB0	0.8803	0.9900	0.3932	0.2606
InceptionResNetV2	0.5980	0.8366	1.0259	1.0107
InceptionV3	0.8564	0.9662	0.3524	0.5360
ResNet50	0.9473	0.9946	0.0627	0.1874
ResNet101	0.9425	0.9914	0.0334	0.3252
ResNet152	0.8755	0.9915	0.0990	0.2349
Shamrat et.al [31]	0.8984	0.9236	0.3897	0.2706
Helaly et.al [30]	0.9123	0.9836	0.2698	0.1896
Yousry et. al [29]	0.9535	0.0.9946	0.0624	0.1963
BEiT	**1**.**0**	**0**.**9841**	**0**.**05**	**0**.**06**

### Comparison with machine learning algorithms

**Table 6:** Results of ML algorithms on OASIS dataset

Method	Accuracy	Specificity	Sensitivity	FNR	FPR
XGBoost	0.8902	0.9633	0.8905	0.1094	0.0366
SVM	0.9132	0.9107	0.9135	0.0864	0.0893
KNN	**0**.**9371**	**0**.**9790**	**0**.**9377**	**0**.**0622**	**0**.**0209**
LR	0.907	0.969	0.9074	0.0925	0.0309
BEiT [10]	**0**.**9843**	**0**.**9931**	**0**.**9696**	**0**.**0303**	**0**.**0068**

Computer vision tasks have seen the application of deep learning to almost any task and have achieved reasonable success on that front. However, not every problem needs to be solved by a deep learning algorithm, which necessitates the availability of adequate samples. Recently, Shaffi et al. [5] have shown that a small number of diverse ML classifiers outperform DL algorithms and also concluded that ML algorithms are the preferred choice over DL algorithms in data-constrained applications such as the medical sector. In this section, we provide the performance of four top-performing ML algorithms from the study conducted by Shaffi et al. [5], which are Support Vector Machines (SVM), K-Nearest Neighbor (KNN), Logistic Regression (LR), and Extreme Gradient Boosting (XGB) [32]. This study will also help us see if computationally and memory-intensive VT architectures are viable, especially when ML algorithms can perform better or on par with contemporary deep learning algorithms. The reported ML results are after determining optimal hyperparameter values based on the grid-search approach using a five-fold cross-validation technique.

**Table 7:** Results of ML algorithms on ADNI dataset

Method	Accuracy	Specificity	Sensitivity	FNR	FPR
XGBoost	0.9809	0.9925	0.9803	0.0196	0.0074
SVM	**0**.**9904**	**0**.**9967**	**0**.**9895**	**0**.**0104**	**0**.**0032**
KNN	0.9809	0.9931	0.9803	0.0196	0.0068
LR	0.9809	0.9925	0.9803	0.0196	0.0074
Swin	0.9952	0.9963	0.9887	0.0112	0.0036

**Fig. 6:**
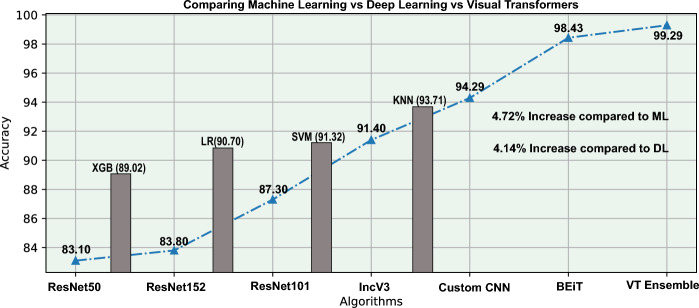
Transformer performance in comparison to ML and DL algorithms

The results of ML algorithms using OASIS and ADNI datasets are tabulated in Table 7 and Table 6, respectively. The KNN algorithm outperformed other ML algorithms on the OASIS dataset (0.9371 acc). Unlike other machine learning algorithms, KNN does not assume any underlying data distribution. This can make it more flexible and fit complex relationships between the input features and target variables. Additionally, KNN can capture local patterns well in the data by assigning a new data point to the class of most of its k-nearest neighbours in the training set [33]. On the other hand, the SVM is particularly well-suited to handle high-dimensional data, where the number of features is vast compared to the number of observations [34]. SVM can use kernel functions to transform the input data into a higher-dimensional space, where it may be possible to find a linear boundary that separates the classes [35]. This allows SVM to handle non-linearly separable data, which may not be possible for other linear models. In addition, SVM has good generalization capabilities on new and unseen data. The SVM algorithm obtained higher recognition accuracy on both OASIS(0.9132) and ADNI (0.9904) datasets.

From Table 7, it can be observed that both the logistic regression and KNN algorithms have the same accuracy score. The KNN has a slightly higher specificity score than logistic regression indicating KNN might better identify negative cases than logistic regression [33]. Logistic Regression performance was better with ADNI compared to OASIS. As the smaller ADNI dataset is imbalanced, the model might have learned to favour the majority class resulting in overall high accuracy. On the other hand, on the larger balanced OASIS dataset, the model might have performed better on all classes but with slightly lower overall accuracy. The XGBoost algorithm performed consistently well on both datasets. It is due to the in-built L1- and L2-regularization techniques which can circumvent situations of overfitting. The regularization techniques are very useful on smaller datasets such as ADNI, where an algorithm can easily overfit. XGB is also scalable to handle large datasets. Compared to ML algorithms, the accuracy and other metrics of VT architectures are significantly higher. There is a nearly 5% increase in the accuracy observed by VT architectures. That is further enhanced when the VT ensembles are utilized. This experiment confirms that the Vision Transformer architectures are very efficient due to their inherent computation of self-attention mechanism that helps to capture long-range dependencies in the MRI images, which in turn helps in gaining overall accuracy.

**Table 8:** Performance comparision of ML, CNN and VT using OASIS dataset

Type.of Models	Methods	Accuracy	Specificity	Sensitivity	FNR	FPR
ML	XGBoost	0.8902	0.9633	0.8905	0.1094	0.0366
	SVM	0.9132	0.9107	0.9135	0.0864	0.0893
	KNN	0.9371	0.9790	0.9377	0.0622	0.0209
	LR	0.907	0.9690	0.9074	0.0925	0.0309
CNN	EfficientNetB0	0.4380	0.5281	0.4121	0.7623	0.331
	InceptionResNetV2	0.5210	0.7655	0.2714	0.7285	0.2344
	ResNet50	0.8492	0.9333	0.8301	0.1698	0.0666
	ResNet101	0.8296	0.9260	0.8013	0.1986	0.0739
	ResNet152	0.8554	0.9382	0.8233	0.1766	0.0617
VT	ViT	0.9718	0.9882	0.9195	0.0808	0.0118
	Swin	0.9804	0.9913	0.9415	0.0584	0.0086
	DeiT	0.9843	0.9931	0.9696	0.0303	0.0068
	BEiT	0.9773	0.9905	0.9533	0.0466	0.0094
VT Ensemble	**BEiT+DeiT (Soft)**	**0**.**9906**	**0**.**9958**	**0**.**9721**	**0**.**0270**	**0**.**0041**
	**Swin+BEiT+DeiT (Hard)**	**0**.**9929**	**0**.**9968**	**0**.**9754**	**0**.**0245**	**0**.**0031**

The performance evaluation of various models are shown in Table 8. It is clearly evident that the VT models significantly outperform CNN and traditional ML algorithms. VT models such as DeiT and BEiT achieve the highest accuracy and sensitivity, with DeiT reaching 98.43% accuracy and 96.96% sensitivity. Ensemble methods further enhance performance, with the Swin+BEiT+DeiT ensemble (hard voting) attaining an exceptional accuracy of 99.29% and sensitivity of 97.54% while maintaining low false negative and false positive rates. These results emphasize the superior ability of VT models to fine-tune effectively for AD classification tasks.

Furthermore, Fig. 6 shows the overall performance improvement of VTs compared with ML and CNN algorithms on the OASIS dataset. There is a significant improvement in accuracy of approximately 5% compared to state-of-the-art ML and CNN models. These set of experiments made us conclude that VTs are efficient classifiers in the four-way classification of AD, whether applied individually or as an ensemble, under varying data conditions. However, the final choice of suitable classifier for real-time application can depend on several parameters and availability of resources. Table 9 shows various circumstances for the choice of classifiers between machine learning, CNN and VTs.

**Fig. 7:**
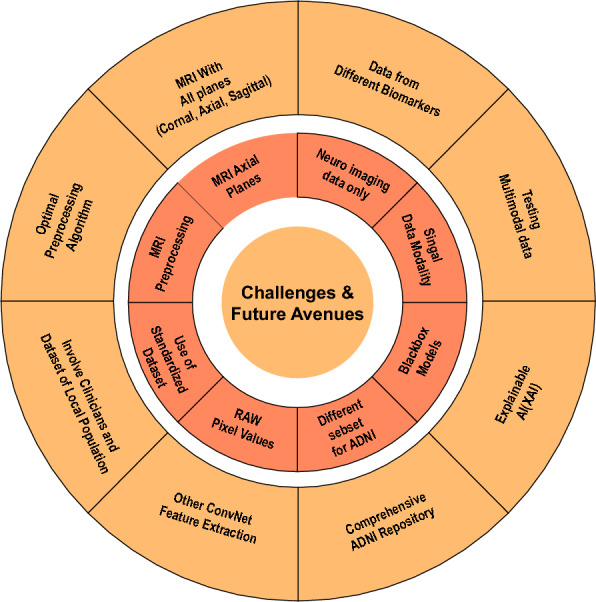
Challenges and Future Avenues

**Table 9:** Choice of classifiers

Parameters	Classical ML	CNN	Transformers
Datatype	ML classifiers are applicable to any dataset, namely, numeric, categorical, text, image, time-series, tabular, and audio. For instance, the medical domain contains images, 1D Data (EEG, ECG etc), neurophysiological measurements, etc.	CNNs are primarily used to classify images. Their architecture is designed specifically for image data, making them particularly effective in image related work.	The transformer architecture is designed to use any dataset. For instance, text data is used in NLP, and images in VT. The transformer encoder eventually converts all datasets into vectors.
Hyperparameter	ML models have fewer hyperparameters that include regularization strength, learning rate, batch size, number of epochs, architecture-specific parameters like layers and units, optimization algorithm, loss function, feature selection, and other model-specific parameters.	CNN have large hyperparameter space that depends on a varying number of convolutional layers, the size and number of filters, the pooling layer size, learning rate, regularization parameters, and many more. Hence, hyperparameter search can be more challenging.	Transformer models depend on dataset-specific hyperparameters like the number of layers, hidden states, attention heads, dropout, learning rate, batch size, and optimizer. Since VT is designed specifically for visual data, hyperparameters include image patch size, number of patches, positional encoding, and input resolution.
Limited Dataset	ML models are better suited as they can be trained using simpler algorithms and few hyperparameters, which can be tuned more efficiently to achieve better performance with simple and reduced computational requirements.	CNN require larger datasets to learn complex patterns in the data effectively. Training a DL model on a limited dataset may lead to overfitting. But CNN can still perform better with smaller datasets by utilizing techniques like data augmentation or regularization to improve performance.	Works well with larger datasets to improve generalization, reduce overfitting, and enhance performance. Specifically, VTs typically require very large datasets to train from scratch. Nevertheless, their performance can remain robust even with limited datasets by fine-tuning a pre-trained model, as demonstrated in this study.
Explainability	ML models are considered more interpretable and are well suited for explaining medical data. ML has a simpler and more interpretable structure, which can make it easier to understand how the model is making predictions. This is because ML models provide clear insights into how individual features contribute to the predictions.	CNN models are typically more complex and difficult to interpret due to their hierarchical and distributed feature representations. Although some techniques like gradient-based attribution methods or saliency maps provide insights into predictions, these methods can be challenging to apply and interpret in practice.	Transformer models offer a balance between performance and interoperability. They provide interpretable representations of input data and offer visualization techniques to understand attention weights. VTs leverage the transformer architecture, especially for visual data, and due to the attention mechanism, they capture long-range dependencies and offer much better explainability.

## Challenges and future avenues

Our study has substantially contributed to the existing knowledge-base on AD classification. However, several challenges remain, and prospective avenues for future academic investigations need to be contemplated. Figure 7 enumerates the challenges identified along with the avenues for further exploration in this section.

1. **Unexplored Preprocessing steps:** MRI scans can be affected by various types of noise, artifacts, and inconsistencies, which can significantly reduce the quality and interoperability of the data. Therefore, proper MRI preprocessing techniques are important to ensure accuracy and reliability of data thereby improving the performance of AI algorithms and provide accurate and consistent results. In this work, MRI images are preprocessed as described in section 3.1. However there exists several other preprocessing factors such as noise reduction, bias field correction, intensity normalization, and other methods to detect and remove artifacts. These preprocessing steps have not been utilized in our study and therefore opens avenues to explore its effect down the proposed pipeline to enable more accurate and meaningful analyses.
2. **Using Only MRI Axial plane:** Relying only on MRI axial planes for AI analysis is one of the biases and poses several challenges, like overlooking pathological features visible in other orientations, like sagittal and coronal planes. This could reduce the sensitivity and specificity of AI models for disease detection and impact diagnostic accuracy. Therefore, there are future prospects for evolving AI algorithms capable of integrating information from multiple image planes to enhance diagnostic accuracy.
3. **Used only neuroimaging data:** The sole use of neuroimaging data poses several challenges. For instance, the data may not capture the full complexity of AD pathology, leading to biased feature representations. Clinicians currently rely on a combination of various biomarkers to assess the likelihood of AD. In our study, we focused solely on MRI data for AD classification. Therefore by integrating multi-modal neuroimaging biomarkers, we could enhance model robustness and diagnostic accuracy. These models provide information on how the predictions were made and which features were critical in making the predictions thereby useful in creating explainable DL models. By doing so, they increase trust and promote the clinical use of ML and DL-based diagnostic tools for AD.
4. **Time-memory overhead**: Although ensembles of VT models perform well in terms of accuracy and other performance metrics, they significantly increase computational resources. Each model needs to be trained on a full dataset, which is time-consuming and resource-intensive. Even if they are trained in parallel mode, computational resources increase. In addition, hyperparameter tuning, such as grid search, for individual models adds additional layers of computational time.
  In terms of memory consumption, each model in the ensemble has its own set of parameters, increasing overall memory usage. Transformer models are known for their large number of parameters. Storing the weights and configurations of each model requires additional memory. This can be substantial, especially when dealing with multiple large models, as conducted in our study. In the future, one can work on optimization or approximation techniques to reduce these overheads.
5. **Blackbox Models: **The models that we have employed for AD classification in this study are all blackbox in nature. We have not fully utilized the self-attention mechanism of VTs and their applicability in interpreting the fine-tuned VT models. In our future endeavors, we aim to fully utilize the inbuilt attention mechanism of VTs and provide attention maps of brain regions that characterize distinct stages of AD. This aligns with the broader objective of enhancing the transparency and interpretability of AI models, facilitating a deeper understanding of the underlying decision-making processes [36, 37].
6. **Different Subsets of ADNI:** In our study, only a subset of MRI images were extracted from the ADNI repository encompassing both male and female subjects aged between 50 and 65 years leading to selection bias. This approach results in generalizability, risks missing important features, and aggravating class imbalances, leading to incorrect predictions. Furthermore, restricting the data may hinder the model from learning from various imaging techniques and long-term data, which is essential for accurate analysis of disease progression. Insufficient representation of the whole dataset can also artificially inflate performance metrics, leading to a misleading sense of accuracy. To address these issues, it is crucial to ensure that the subset is representative, employ cross-validation, maintain a balanced dataset, and transparently report limitations to improve the model’s robustness and applicability.
7. **Raw Pixel Values Used For Training:** In our study we trained all models (ML, DL, VT) using direct raw pixel values. Instead of raw pixel values, we can extract robust low-dimensional features and use them for training the model. This deserves a thorough independent study as a number of different features space can be used such as subspace algorithms like Principal Component Analysis (PCA), Fisher Linear Discriminant Analysis (FLDA), frequency domain features such as Wavelets, Discrete Cosine Transform, etc. One can even study application of various pretrained ConvNet architectures for feature extraction and subsequent model training.
8. **Standard datasets:** In this work, we used standardized datasets, ADNI and OASIS. However, relying solely on standardized datasets may limit the diversity and representativeness of the data, potentially overlooking unique characteristics or differences present in specific patient populations. On the contrary, incorporating proprietary datasets for AD enhances the viability of AI tools in disease care by enriching the dataset with specialized or targeted information. Proprietary datasets are privately owned and not freely accessible. These datasets may contain data from niche populations with specific clinical settings that are not found in standardized datasets. By leveraging these additional data sources, along with the expertise of clinicians to identify clinically relevant patterns or features and interpret and validate the data, we can develop more robust and accurate diagnostic models for conditions like Alzheimer’s disease. This dataset can, therefore, serve as a valuable resource for advancing knowledge, improving care delivery, and addressing the healthcare needs of a niche population.

## Conclusion

Vision Transformer architectures have emerged as an alternative to traditional CNN-centric models for computer vision tasks. Their ability to discover dependency among image patches through a multi-head attention mechanism has enabled the VTs to demonstrate higher accuracy consistently across several benchmark datasets.

In this work, we proposed an ensemble of VT architectures for the task of AD classification. The ensemble VTs demonstrated a 2% improvement over individual VT models. We used both soft and hard voting mechanisms for fusion, where the soft ensemble of BEiT and DeiT models obtained an accuracy of 99.06% while the hard ensemble of Swin, BEiT, and DeiT obtained the highest accuracy of 99.29%. The research also involved a comparative analysis of VTs versus ML and CNN algorithms using the ADNI and OASIS datasets. We observed a significant increase of approximately 5% in accuracy for VTs compared to traditional ML and CNN models, showcasing VTs’ exceptional fine-tuning capability even with limited samples. We also identified and addressed several limitations in our research work, paving the way for future studies to build upon our findings and further enhance the effectiveness of Vision Transformer architectures in Alzheimer’s Disease classification.

## Data Availability

The codes used in this study are available in the Github page: https://github.com/snoushath/VIT-CNN-AD.
